# Supplementary material for: Factors associated with HIV testing among young females; further analysis of the 2016 Ethiopian demographic and health survey data
Source: PLoS One. 2020 Feb 11;15(2):e0228783. doi: 10.1371/journal.pone.0228783 (PMC7012428; doi:10.1371/journal.pone.0228783)
Supplement: Attachment — Submitted filename: Summary for reviewers comments.docx [file pone.0228783.s001.docx]

**Factors associated with HIV testing among young females; further analysis of the 2016 Ethiopian Demographic and Health Survey.**

# SUMMARISED COMMENTS

| **Section** | **Comment, question, suggestion.** |
| --- | --- |
| **Abstract** | 1. Well summarised abstract. 2. Lacked quantifications of the problem, how low is low HIV testing in Ethiopia? 3. Regarding the knowledge gap:    - What are the advantages of us having this information? Who will benefit from the study being conducted and how?    - Limited information does not warrant a study to be conducted. The missing information has to be useful in some way to the study stakeholders. 4. Would be better to mention the age range. Who is considered to be a young woman for this study? 15-19 years? Or 15-24? 5. The conclusion summarized the results without any further recommendations.    - Recommendations are needed to show the implication of the study findings either to policy and/or to practice. |
| **Introduction**  **Background** | 1. Throughout the entire background, entire paragraphs had only one citation on the last sentence. Please revisit citations.  2. It is still unclear who is a “young woman”. It is informative for the reader to have an age range earlier on in the paper.  3. For the paragraph concerning previously identified significant predictors;  It would be more informative to mention the exact levels/categories which were found to be significant predictors e.g. Rural/urban? Which category was significant?  It would also be informative to mention whether they are predictors of higher/lower odds of HIV testing.  4. And thereafter identifying the factors, how would the results be used? After these bottle-necks have been identified. |
| **Methodology** | 1. Why only include women who were sexually active in the past 12 months into the study? Please provide an elaboration.    - The risk of HIV does not change regardless of the time since last sexual intercourse.   Wouldn’t a more appropriate exclusion criteria be women who NEVER had sexual intercourse?  2. For the outcome definition;  Why the focus on those who tested in past 12 months?  Why not use if she EVER tested for HIV?  Why are those who tested before 12 months considered as if they have never tested and coded as 0?  3. How was HIV knowledge measured?  4. Did you consider using alternatives to logistic regression if you identified the outcome of interest to be common? >10% prevalent.  5. “Bivariate regression analysis was done to examine associations between contraceptive use and the selected predictor variables”;  Does this study measure contraceptive use OR HIV testing?  6. “In addition, correlation among predictor variables was assed using variance inflation factor before recruiting variables to the final mode.”  What do you mean? Is it correlation or collinearity? |
| **Results** | 1. For every table caption, please include N=???  2. For every variable in the tables with category “other”, please provide an explanation on who/what “other” means.  3. Did you consider life time sexual partners? Or those in the past 12 months? Please make clear.  4. What is chat? Concerning the variable “chewed chat”  5. The percentages presented in Table 3 are column percentages,  Which means the interpretations should be “among those tested for HIV, 26.22% were aged 15-19”.  Please revise the interpretation provided above. |
| **Discussion** | Please apply these for the entire discussion section;  1. For each finding, it would be more informative to report numbers/findings that these previous studies reported. It would be informative to mention them in brief so the reader may compare.   2. What is the implication of each study finding? Are you recommending for the Ethiopian government to adopt a similar strategy to increase HIV testing as Uganda?   3. Discussing only similarities/differences of study findings is not enough. You need to go a step further and show the implications of this study findings to either policy or to practice. |
| **Strengths and limitations** | 1. This section is missing from the paper |
| **Conclusion + Recommendations** | 1. The conclusion provided a summary of the main results of the paper. 2. Please provide recommendations basing on the results of this study. How can these findings be applied to benefit the stakeholders of the study? The women, policy makers, government officials? |
