# Supplementary material for: Factors associated with HIV testing among young females; further analysis of the 2016 Ethiopian demographic and health survey data
Source: PLoS One. 2020 Feb 11;15(2):e0228783. doi: 10.1371/journal.pone.0228783 (PMC7012428; doi:10.1371/journal.pone.0228783)
Supplement: Attachment — Submitted filename: Mahande_PONE-D-19-32104_MASHA_edit.docx [file pone.0228783.s002.docx]

**PLOS ONE**

**Factors associated with HIV testing among young females; further analysis of the 2016 Ethiopian Demographic and Health Survey.**

|  |  |  |  |  | --Manuscript Draft-- |
| --- | --- | --- | --- | --- | --- |
|  |  |  |  |  |  |
| **Manuscript Number:** | | | | | PONE-D-19-32104 |
| **Article Type:** | | | | | Research Article |
|  |  |  |  |  |  |
| **Full Title:** | | | | | Factors associated with HIV testing among young females; further analysis of the 2016 |
|  |  |  |  |  | Ethiopian Demographic and Health Survey. |
| **Short Title:** | | | | | Factors associated with HIV testing among young females |
|  |  |  |  |  |  |
| **Corresponding Author:** | | | | | Yibeltal Alemu Bekele, MpH |
|  |  |  |  |  | Bahir Dar University College of Medical and Health Sciences |
|  |  |  |  |  | Bahir Dar, ETHIOPIA |
| **Keywords:** | | | | | HIV testing, young women, Ethiopia, Ethiopian Demographic health survey |
|  |  |  |  |  |  |
| **Abstract:** | | | | | Background: HIV counseling and testing is key to control and prevent the spread of the |
|  |  |  |  |  | virus and improve the life of people living with HIV. Although the risk of acquiring the |
|  |  |  |  |  | virus is high, HIV testing and counseling service use is low among young women in |
|  |  |  |  |  | Ethiopia. There are few studies about factors associated with HIV testing and |
|  |  |  |  |  | counseling service use among young females in Ethiopia. Therefore, this analysis was |
|  |  |  |  |  | done to fill this gap. |
|  |  |  |  |  | Methods: The study used the 2016 Ethiopian demographic and health survey data. |
|  |  |  |  |  | The data was downloaded from The DHS program with permission. A total of 2661 |
|  |  |  |  |  | young women were included in the final model. Data was weighted to consider |
|  |  |  |  |  | disproportionate sampling and non-response. Complex data management technique |
|  |  |  |  |  | was applied to consider the complex sampling technique used in the DHS. |
|  |  |  |  |  | Multivariable logistic regression was used to identify factors associated with HIV testing |
|  |  |  |  |  | among young women. |
|  |  |  |  |  | Result: Among sexually active young women, 33.5 % (95%CI; 30.1, 37.1) were tested |
|  |  |  |  |  | for HIV. Young women who attended primary ((AOR 2.78; (95% CI; 2.01, 3.87)), |
|  |  |  |  |  | secondary (AOR 4.73; (95% CI; 3.07, 7.29)) and higher education (AOR; 5.55 95% CI; |
|  |  |  |  |  | 2.56, 12.02), young women who had multiple sexual partners (AOR 5.49; 95% (CI; |
|  |  |  |  |  | 1.29, 23.27)), those who ever had alcohol (AOR 1.46; 95% (CI; 1.09, 1.98)) and young |
|  |  |  |  |  | women who visited health facility (AOR 1.78; (95% CI; 1.36, 2.32)) higher odds of |
|  |  |  |  |  | being tested for HIV. On the other hand, young women from the rural area had lower |
|  |  |  |  |  | odds (AOR 0.47; (95% CI; 0.31, 0.72)) being tested. |
|  |  |  |  |  | Conclusion: HIV testing among sexually active young women in Ethiopia is low. |
|  |  |  |  |  | Educational status, place of residence, history of alcohol intake, numbers of sexual |
|  |  |  |  |  | partner and visiting health facility 12 months before the survey were found significant |
|  |  |  |  |  | predictors of HIV testing among sexually active young women in Ethiopia. |
| **Order of Authors:** | | | | | Yibeltal Alemu Bekele, MpH |
|  |  |  |  |  |  |
|  |  |  |  |  | Gedefaw Abeje Fekadu |
|  |  |  |  |  |  |
| **Additional Information:** | | | | |  |
| **Question** | | | | | **Response** |
|  |  |  |  |  |  |
| **Financial Disclosure** | | | | | The authors received no specific funding for this work. |
| Enter a financial disclosure statement that | | | | |  |
| describes the sources of funding for the | | | | |  |
| work included in this submission. Review | | | | |  |
| the [submission guidelines](http://journals.plos.org/plosone/s/submission-guidelines#loc-financial-disclosure-statement) for detailed | | | | |  |
|  |  |  |  |  |  |
| requirements. View published research | | | | |  |
| articles from [*PLOS ONE*](http://journals.plos.org/plosone/) for specific | | | | |  |
| examples. | |  |  | |  |
|  |  |  |  |  |  |

*Powered by Editorial Manager® and ProduXion Manager® from Aries Systems Corporation*

This statement is required for submission

and **will appear in the published article** if

the submission is accepted. Please make

sure it is accurate.

**Unfunded studies**

Enter: *The author(s) received no specific*

*funding for this work.*

**Funded studies**

Enter a statement with the following details:

- Initials of the authors who received each award
- Grant numbers awarded to each author
- The full name of each funder
- URL of each funder website
- Did the sponsors or funders play any role in the study design, data collection and analysis, decision to publish, or preparation of the manuscript?
- **NO** - Include this sentence at the end of your statement: *The funders had no role in* *study design, data collection and analysis, decision to publish, or preparation of the manuscript.*
- **YES** - Specify the role(s) played.
- typeset

| **Competing Interests** | The authors have declared that no competing interests exist. |
| --- | --- |

Use the instructions below to enter a

competing interest statement for this

submission. On behalf of all authors,

disclose any [competing interests](http://journals.plos.org/plosone/s/competing-interests) that

could be perceived to bias this

work—acknowledging all financial support

and any other relevant financial or non-

financial competing interests.

This statement **will appear in the**

**published article** if the submission is

accepted. Please make sure it is

accurate. View published research articles

from [*PLOS ONE*](http://journals.plos.org/plosone/) for specific examples.

*Powered by Editorial Manager® and ProduXion Manager® from Aries Systems Corporation*

**NO authors have competing interests**

Enter: *The authors have declared that no*

*competing interests exist.*

**Authors with competing interests**

Enter competing interest details beginning

with this statement:

*I have read the journal's policy and the*

*authors of this manuscript have the following*

*competing interests: [insert competing*

*interests here]*

| * typeset | | |  |
| --- | --- | --- | --- |
|  |  |  |  |
| **Ethics Statement** | | | The 2016 EDHS protocol was reviewed and approved by the National Ethics Review |
|  |  |  | Committee of the Federal Democratic Republic of Ethiopia, Ministry of Science and |
| Enter an ethics statement for this | | | Technology and the Institutional Review Board of ICF International. |
| submission. This statement is required if | | |  |
| the study involved: | | |  |
| • Human participants | | |  |
| • Human specimens or tissue | | |  |
| • Vertebrate animals or cephalopods | | |  |
| • Vertebrate embryos or tissues | | |  |
| • Field research | | |  |
| Write "N/A" if the submission does not | | |  |
| require an ethics statement. | | |  |
| General guidance is provided below. | | |  |
| Consult the [submission guidelines](http://journals.plos.org/plosone/s/submission-guidelines#loc-guidelines-for-specific-study-types) for | | |  |
|  |  |  |  |
| detailed instructions. **Make sure that all** | | |  |

**information entered here is included in the**

**Methods section of the manuscript.**

*Powered by Editorial Manager® and ProduXion Manager® from Aries Systems Corporation*

**Format for specific study types**

**Human Subject Research (involving human**

**participants and/or tissue)**

- Give the name of the institutional review board or ethics committee that approved the study
- Include the approval number and/or a statement indicating approval of this research
- Indicate the form of consent obtained (written/oral) or the reason that consent was not obtained (e.g. the data were analyzed anonymously)

**Animal Research (involving vertebrate**

**animals, embryos or tissues)**

- Provide the name of the Institutional Animal Care and Use Committee (IACUC) or other relevant ethics board that reviewed the study protocol, and indicate whether they approved this research or granted a formal waiver of ethical approval
- Include an approval number if one was obtained
- If the study involved *non-human primates*, add *additional details* about animal welfare and steps taken to ameliorate suffering
- If anesthesia, euthanasia, or any kind of animal sacrifice is part of the study, include briefly which substances and/or methods were applied

**Field Research**

Include the following details if this study

involves the collection of plant, animal, or

other materials from a natural setting:

- Field permit number
- Name of the institution or relevant body that granted permission

| **Data Availability** | Yes - all data are fully available without restriction |
| --- | --- |

Authors are required to make all data

underlying the findings described fully

available, without restriction, and from the

time of publication. PLOS allows rare

exceptions to address legal and ethical

concerns. See the [PLOS Data Policy](http://journals.plos.org/plosone/s/data-availability) and

[FAQ](http://journals.plos.org/plosone/s/data-availability#loc-faqs-for-data-policy) for detailed information.

*Powered by Editorial Manager® and ProduXion Manager® from Aries Systems Corporation*

A Data Availability Statement describing

where the data can be found is required at

submission. Your answers to this question

constitute the Data Availability Statement

and **will be published in the article**, if

accepted.

**Important:** Stating ‘data available on request

from the author’ is not sufficient. If your data

are only available upon request, select ‘No’ for

the first question and explain your exceptional

situation in the text box.

Do the authors confirm that all data

underlying the findings described in their

manuscript are fully available without

restriction?

**Describe where the data may be found in**	All relevant data are within the manuscript and its Supporting Information files.

**full sentences. If you are copying our**

**sample text, replace any instances of XXX**

**with the appropriate details.**

- If the data are **held or will be held in a** **public repository**, include URLs, accession numbers or DOIs. If this information will only be available after acceptance, indicate this by ticking the box below. For example: *All* *XXX* *files* *are available from the XXX database (accession number(s) XXX, XXX.*).
- If the data are all contained **within the** **manuscript and/or Supporting Information files**, enter the following: *All relevant data are within the manuscript and its Supporting Information files.*
- If neither of these applies but you are able to provide **details of access** **elsewhere**, with or without limitations, please do so. For example:

*Data cannot be shared publicly because of [XXX]. Data are available from the XXX Institutional Data Access / Ethics Committee (contact via XXX) for researchers who meet the criteria for access to confidential data.*

*The data underlying the results*

*presented in the study are available*

*from (include the name of the third party*

*Powered by Editorial Manager® and ProduXion Manager® from Aries Systems Corporation*

*and contact information or URL).*

- This text is appropriate if the data are owned by a third party and authors do not have permission to share the data.

* typeset

Additional data availability information:

*Powered by Editorial Manager® and ProduXion Manager® from Aries Systems Corporation*

Manuscript

[Click here to download Manuscript HIV testing among young](https://www.editorialmanager.com/pone/download.aspx?id=25545408&guid=8d101a49-9035-42ea-b029-16644ee9d242&scheme=1) [Girls new.docx](https://www.editorialmanager.com/pone/download.aspx?id=25545408&guid=8d101a49-9035-42ea-b029-16644ee9d242&scheme=1)

Factors associated with HIV testing among young females; further analysis of the 2016 Ethiopian Demographic and Health Survey.

Yibeltal Alemu Bekele^1*^ and Gedefaw Abeje Fekadu^1^

Authors’ email address

YAB [yibeltalalemu6@gmail.com](mailto:yibeltalalemu6@gmail.com)

GAF [abejegedefaw@gmail.com](mailto:abejegedefaw@gmail.com)

*correspondent Author

^1^Department of Reproductive Health and Population Studies, School of Public Health, Bahir Dar University, Bahir Dar, Ethiopia

**Abstract**

**Background:** HIV counseling and testing is key to control and prevent the spread of the virus and improve the life of people living with HIV. Although the risk of acquiring the virus is high, HIV testing and counseling service use is low among young women in Ethiopia. There are few studies about factors associated with HIV testing and counseling service use among young females in Ethiopia. Therefore, this analysis was done to fill this gap.

**Methods:** The study used the 2016 Ethiopian demographic and health survey data. The data was downloaded from The DHS program with permission. A total of 2661 young women were included in the final model. Data was weighted to consider disproportionate sampling and non-response. Complex data management technique was applied to consider the complex sampling technique used in the DHS. Multivariable logistic regression was used to identify factors associated with HIV testing among young women.

**Result:** Among sexually active young women, 33.5 % (95%CI; 30.1, 37.1) were tested for HIV. Young women who attended primary ((AOR 2.78; (95% CI; 2.01, 3.87)), secondary (AOR 4.73; (95% CI; 3.07, 7.29)) and higher education (AOR; 5.55 95% CI; 2.56, 12.02), young women who had multiple sexual partners (AOR 5.49; 95% (CI; 1.29, 23.27)), those who ever had alcohol (AOR 1.46; 95% (CI; 1.09, 1.98)) and young women who visited health facility (AOR 1.78; (95% CI;

1.36, 2.32)) higher odds of being tested for HIV. On the other hand, young women from the rural area had lower odds (AOR 0.47; (95% CI; 0.31, 0.72)) being tested.

**Conclusion:** HIV testing among sexually active young women in Ethiopia is low. Educational status, place of residence, history of alcohol intake, numbers of sexual partner and visiting health facility 12 months before the survey were found significant predictors of HIV testing among sexually active young women in Ethiopia.

Keywords, HIV testing, young women, Ethiopia, Ethiopian Demographic health survey

**Background**

Globally 36.5 million people were living with HIV. Every day 5,000 people were infected with the virus. The Eastern and Southern Africa remains the most affected region accounting for 53% of people living with HIV. Young people are disproportionally affected by HIV. Among people living with HIV, 30% are young. Young women accounted for 64% of young people living with HIV. The problem is more sever in Sub-Sahara Africa(1-3).

Young people are exposed to HIV at two points of their lives; at the early age of life transmitted from mother-to-child and during their adolescent time as a result of their sexual behaviors and gender disparities. Young women and girls are disproportionally affected by HIV because of economic, cultural and social disparity in the society (1, 4-8).

HIV counseling and testing services are essential for HIV prevention, treatment, care, and support. In 2016, United Nations declared to end AIDS at the end of 2020.The declaration endorsed the 90-90-90 target. Increasing access to and uptakes of HIV testing is critical to achieve this target. However, around 30 % of people living with HIV were not aware of their HIV status(9-11).

The coverage of HIV testing and counseling in SSA remained considerably low, especially among young women. Studies in Nigeria, Rwanda, Uganda and Kenya indicated that only 12%, 40%, 26.5% 27.7% young women were tested for HIV respectively (12-15).

Studies conducted in different parts of the globe identified age, place of residence, work status, educational status, knowledge on HIV, discussion about HIV with mother or female guardian, history of pregnancy, age at first sexual debut, wealth index, marital status, number of sexual partner, history of sexually transmitted infection and history of antenatal care as determinants of HIV testing(16-25).

Ethiopia is one of the Sub-Saharan countries affected with HIV. The government is committed to end the epidemic at the end of 2030. In 2010, the ministry of health set an ambitious goal to end new infections among newborn children at the end of 2020. But the HIV prevalence rate increased by 10% from 0.3/1000 population in 2008 to 0.33/1000 population in 2016. HIV counseling and testing was one of the strategies designed to achieve the 2020 goal. But, according to the 2016

Ethiopian demographic health survey report, indicated that only 36.4% young women were tested for HIV (11, 26-29).

There are few studies about factors associated with HIV testing among young women in Ethiopia. Identifying determinants will help identify the bottlenecks for HIV testing service use. Therefore, this analysis was done to identify factors associated with HIV testing among young women in Ethiopia.

**Method and materials**

**Data**

The 2016 Ethiopian demographic and health survey (EDHS) data was used for this analysis. The data was downloaded from The DHS program after permission. The 2016 EDHS is a community based, nationally representative data collected from January 18, 2016, to June 27, 2016.It was implemented by the Central Statistics Authority (CSA) and ICF international. EDHS followed two stage stratified sampling technique. A total of 15,683 reproductive age (15 to 49 years old) women were included in the survey. From this 6,401 were aged 15 to 24 years. Among these women, 2,661 had history of sexual intercourse within 12 months before the survey. Only these women were included in the survey.

**Variables**

The outcome variable for this study was HIV testing; a dichotomous variable coded “1”if a young woman reported that she was tested for HIV in the last 12 months and “0” if she reported otherwise (never tested or tested before 12 months). All reproductive age women included in the survey were asked whether they were tested for HIV or not. Therefore, the dependent variable was generated from this question.

The independent variables were categorized to two groups; socio-demographic and behavioral. The socio-demographic variables were age, religion, residence, wealth index, educational status, marital status and work status at the time of survey.

Behavior related variables were HIV knowledge, discussion about HIV with mother or female guardian, history of pregnancy, age at first sexual debut, history of sexually transmitted infection, number of sexual partners, substance use and history of antenatal care.

**Statistical analysis**

Data analysis was done using STATA 15.1. After the data set was downloaded from The DHS program, the dependent and independent variables were identified. First, descriptive analysis of each variable was done. Bivariate regression analysis was done to examine associations between contraceptive use and the selected predictor variables. Variables were included into the multivariate logistic regression model if they had a significant association at the bivariate level (P-value <0.05). In addition, correlation among predictor variables was assed using variance inflation factor before recruiting variables to the final mode. Multivariable logistic regression analysis was used to identify factors associated with the outcome variable after adjusting for potential confounders. We weighted the data when computing proportions to consider the non-response and disproportionate sampling in DHS sampling process. Since DHS used complex sampling, this was considered in the analysis.

**Result**

A total of 2661 young, sexually active women were included in the final model. One thousand eight hundred ninety eight (73.33%) of the respondents were aged 20 to 24 years. One thousand two hundred thirteen (45.58%) of the respondents were Muslims in terms of religion. One thousand one hundred eighty eight (44.64%) of the respondents did attained primary education. Two thousand three hundred fifteen (86.99%) of the respondents were married. One thousand nine hundred twelve (75.85%) of the respondents were not working at the time of survey. One thousand nine hundred thirty (72.53%) of the respondents were rural residents (Table 1).

Table 1. Socio-demographic characteristic of young, sexually active women survey Ethiopia, EDHS 2016

| **Characteristics** | **Number (%)** |
| --- | --- |
|  |  |
| **Age** |  |
| 15 to 19 | 763 (28.67) |
| 20 to 24 | 1898(73.33) |
|  |  |
| **Religion** |  |
| Orthodox | 985(37.02) |
| Muslim | 1213(45.58) |
| Other | 463(17.39) |
| **Educational status** |  |
| No education | 894(33.59) |

| Primary | 1188(44.64) |
| --- | --- |
| Secondary | 405(15.22) |
| Higher | 174(6.54) |
| **Marital status** |  |
| Single | 187(7.03) |
| Married or living in union | 2315(86.99) |
| Other | 159(5.98) |
| **Age at first birth** |  |
| Before 20 years | 1315(76.23) |
| At 20 and after years | 410(23.77) |
| **Currently working** |  |
| No | 1912(71.85) |
| Yes | 355(28.15) |
| **Wealth index** |  |
| Poorest | 813(30.55) |
| Poor | 383(14.39) |
| Middle | 345(12.97) |
| Richer | 312(11.72) |
| Richest | 808(30.36) |
| **Residence** |  |
| Urban | 731(27.47) |
| Rural | 1930(72.53) |

**Behavioral characteristics of young sexually active women**

Two thousand three hundred sixty eight (88.99%) of the respondents reported that they started sexual intercourse before the age of 20. Three hundred thirteen (11.76%) respondents reported that their most recent sex was with non-spousal. One thousand four hundred three (52.72%) respondents visited health facility twelve 12 months before the survey. Eight hundred seven (30.33%) of the respondents were ever drunk alcohol. Twelve (3.48%) of the respondents had sex in return for gifts and cash (Table 2).

Table 2. Behavioral characteristics of sexually active young women in Ethiopia, EDHS 2016

| **Characteristics** | **Numbers (%)** |
| --- | --- |
|  |  |
| **Age at first sex** |  |
| Before 20 years | 2368(88.99) |
| At 20 and after years | 293(11.01) |
| **Numbers of sexual partners** |  |
| One | 2636(99.06) |
| More than one | 25(0.94) |
| **Relationship with the most recent** |  |
| **sexual partner** |  |
| Spouse | 2348(88.24) |
| Non-spouse | 313(11.76) |
| **Visited health facility in the last 12** |  |
| **months** |  |
| Yes | 1403(52.72) |
| No | 1258(47.28) |
| **Chewed Chat** |  |
| No | 2426(91.97) |
| Yes | 235(8.83) |
| **Ever drunk alcohol** |  |
| No | 1854(69.67) |
| Yes | 807(30.33) |
| **Had sex in return for gifts, cash or other** |  |
| No |  |
| Yes | 333(96.52) |
|  | 12(3.48) |

**HIV testing**

Among young women who had history of sexual intercourse with in the last 12 month, only 33.5% (95%CI; 30.1, 37.1) were tested for HIV.HIV testing among young women varied across regions. The highest proportions of young women tested were from Amhara region (9.76%) followed by Oromia (9.38 %) and SNNPR (5.74%) regions. On the other hand, the proportion of young women tested for HIV from Harari (0.13%), Gambela (0.2%) and Dire Dewa (0.3%) regions was low.

**HIV testing by women characteristics**

Seven hundred forty (77.78%) respondents aged 20 to 24 years were test for HIV. Eight hundred twenty (81.75%) married respondents were tested for HIV. Four hundred eight one (47.96%) respondents who attained primary education were test for HIV while only 151(15.05%)

respondents who did not attained formal education were tested for HIV. Among urban residents, four hundred sixty-three (46.16%) were tested for HIV (Table 3).

Table 3 Characteristics of HIV tested, sexually active, young women in Ethiopia, EDHS 2016

| Characteristics | Number (%) |
| --- | --- |
|  |  |
| **Age** |  |
| 15 to 19 | 263(26.22) |
| 20 to 24 | 740(77.78) |
| **Religion** |  |
| Orthodox | 505(50.35) |
| Muslim | 327(32.60) |
| Other | 171(17.05) |
| **Educational status** |  |
| No education | 151(15.05) |
| Primary | 481(47.96) |
| Secondary | 246(24.53) |
| Higher | 125(12.46) |
| **Marital status** |  |
| Single | 116(11.57) |
| Married or living in union | 820(81.75) |
| Other | 67(6.68) |
| **Age at first birth** |  |
| Before 20 years | 396(68.99) |
| At 20 and after years | 178(31.01) |
| **Currently working** |  |
| No | 648(64.61) |
| Yes | 355(35.39) |
| **Wealth index** |  |
| Poorest | 125(12.46) |
| Poor | 121(12.06) |
| Middle | 114(11.37) |
| Richer | 136(13.56) |
| Richest | 507(50.55) |
| **Residence** |  |
| Urban | 463(46.16) |
| Rural | 540(53.84) |
| **Age at first sex** |  |
| Before 20 years | 841(83.85) |
| At 20 and after years | 162(16.15) |
| **Visited health facility within 12 months** |  |
| Yes | 662(66.00) |
| No | 341(34.00) |

**Factors associated with HIV testing among sexually active young women**

In the multivariable logistic regression model, number of sexual partners, educational status, marital status, residence, history of health facility visit 12 months before the survey and drinking alcohol were found significantly associated with HIV testing (table4).

The odds of HIV testing among young women who attended primary, secondary and higher education was 2.78(AOR 2.78; (95% CI; 2.01, 3.87)), 4.73 (AOR 4.73; (95% CI; 3.07, 7.29)) and 5.55 (AOR; 5.55 95% CI; 2.56, 12.02) respectively higher compared to those who did not attend formal education. The odds of HIV testing among young women who were widowed, divorced and separated was 2.18 times higher compared to young women who were single (AOR 2.18; (95% CI; 1.01, 4.71)).Young women who were living in rural area had63%(AOR 0.47; (95% CI; 0.31, 0.72)) lower odds to be tested for HIV compared to their urban counter parts (table 4).

The odds of HIV testing among sexually active, young women who had multiple sexual partners were 5.49 (AOR 5.49; 95% (CI; 1.29, 23.27)) times higher compared to young women who had one sexual partner. The odds of HIV testing among young women who have had ever take alcohol was 1.46 (AOR 1.46; 95% (CI; 1.09, 1.98)) times higher compared to those who had not. The odds of HIV testing among young women who visited health facility in the last 12 months was 1.78(AOR 1.78; (95% CI; 1.36, 2.32)) times higher than those young women’s who didn’t (table 4).

Table 4Factors associated with HIV testing among sexually active, young women in Ethiopia, EDHS 2016.

| **Variables** | **HIV test** |  | **COR (95 % CI)** | **AOR (95% CI)** |
| --- | --- | --- | --- | --- |
|  |  |  |  |  |
|  | **Yes** | **No** |  |  |
|  |  |  |  |  |
| **Age** |  |  |  |  |
| 15 to 19 | 263 | 500 | 1 | 1 |
| 20 to 24 | 740 | 1158 | 1.21(0.91,1.61) | 1.14(0.84,1.56) |
| **Educational status** |  |  |  |  |
| Not attaining education | 151 | 743 | 1 | 1 |
| Primary education | 481 | 707 | 2.95(2.13,4.09) | 2.78(1.01,3.86) |
| Secondary education | 246 | 159 | 6.88(4.39,10.77) | 4.73(3.07,7.29) |
| Higher | 125 | 49 | 10.05(5.09,19.84) | 5.55(2.56,12.02) |
| **Age at first sex** |  |  |  |  |
| Before 20 years | 841 | 131 | 1 | 1 |
| At 20 and after | 162 | 162 | 1.87(1.25,2.80) | 1.11(0.96,1.78) |
| **Marital status** |  |  |  |  |
| Single | 116 | 71 | 1 | 1 |
| Married/ in union | 820 | 1495 | 0.45(0.27,0.77) | 1.31(0.74,2.35) |
| Other | 67 | 92 | 0.73(0.34,1.62) | 2.18(1.01,4.71) |
| **Residence** |  |  |  |  |
| Urban | 463 | 268 | 1 | 1 |
| Rural | 540 | 1390 |  | 0.47(0.31,0.72) |
| **Current working status** |  |  |  |  |
| No |  |  |  |  |
| Yes | 648 | 1264 | 1 | 1 |
|  | 355 | 394 | 1.72(1.26,2.35) | 1.12(0.80,1.57) |
| **Numbers of sexual partner** |  |  |  |  |
| One |  |  |  |  |
| More than one | 989 | 1647 | 1 | 1 |
|  | 14 | 11 | 7.44(2.23,24.84) | 5.49(1.29,23.27) |
| **Ever taken alcohol** |  |  |  |  |
| No | 617 | 1237 | 1 | 1 |
| Yes | 386 | 421 | 1.66(1.24,2.23) | 1.46(1.09,1.98) |
| **Visiting health facility in the last** |  |  |  |  |
| **12 months** |  |  |  |  |
| No | 341 | 917 | 1 | 1 |
| Yes | 662 | 741 | 2.03(1.57,2.62) | 1.77(1.36,2.32) |
|  |  |  |  |  |

**Discussion**

The magnitude of HIV testing among young women who were sexually active (had history of sexual intercourse 12 months before the survey) were 33.5% (95%CI; 30.1, 37.1). This finding was consistent with studies conducted in South Africa(30).However this finding was lower than studies conducted Uganda(31) and another studies conducted in South Africa(16). The reason for this difference was that Uganda government used different modality to reach the community for HCT like integrating HCT in to routine health care service at all levels, expansions of community outreach/mobile HCT services targeting the grass root and hard to reach areas; and gave strong emphasis on provider initiated HCT to minimize missed opportunity(32). Uganda is one of a few Sub-Saharan countries effectively implemented home based HCT service that plays a major role in expanding access of HCT(33).

This study identified that young women who attended formal education had more odds of being tested for HIV. This finding was consistent with studies conducted in Tanzania(17, 34) and Nigeria,(24, 25). The reason for this is that education can improve HIV knowledge among people. Education also empowers women to make their decision to visit health facility and use health services. In addition, education improves income among women which in turn increase health service use(35).

Young women who live in a rural area were 53% less likely to be tested for HIV compared to their urban counterparts. Similar conclusion were drawn from studies conducted in Ethiopia(36) and Nigeria(24, 25). The reason for this may be better availability and accessibility of HIV testing facilities in urban settings(37).

Young women with multiple sexual partners were more likely to be tested for HIV compared to those with one lifetime sexual partner. This finding was similar with study conducted in Tanzania 2016(17), Uganda(31) and Thailand(19). This might be due to the fact that women with multiple sexual partners had higher perceived risk of getting HIV. This in turn increases their motive to be tested (38). Moreover, program planners and health professionals may give more emphasis for these women considering them high risk.

Young women who ever drunk alcohol had higher odds of being tested for HIV compared to women who never drank alcohol. This could be due to risky sexual behavior after alcohol. This may have increased perceived susceptibility which leads to HIV testing(39) (40).

This study showed that women who visit health facility 12 months before the survey had more odds to be tested for HIV compared to young women who didn’t visit health facility. This finding was similar with a study conducted in South Africa(30). The reason for this may be due to health professionals initiate people who visited health facilities for HIV testing. The service is provided at all governmental and public health facilities(41). Ethiopia adopted Provider initiated counseling and testing /PICT/ for all outpatient and inpatient clients (41, 42).

**Conclusion**

HIV testing among sexually active young women in Ethiopia is low. Educational status, place of residence, history of alcohol intake, number of sexual partner and visiting health facility 12 months before the survey were found significant predictors of HIV testing among sexually active young women in Ethiopia.

**Declaration**

**Ethics approval and consent to participate**

The 2016 EDHS protocol was reviewed and approved by the National Ethics Review Committee of the Federal Democratic Republic of Ethiopia, Ministry of Science and Technology and the Institutional Review Board of ICF International. The STATA format data was downloaded from the DHS program with permission.

**Consent for publication**

Not applicable

**Availability of data and material**

Upon request, the data is available on The DHS program website at

**Computing Interests**

The authors declare that there is no conflict of interest with regard to the publication.

**Funding**

No external fund was received.

**Author contributions**

Y.A.B initiated the study, performed the analysis and prepared the manuscript. GAF involved equally in the analysis and preparation of the manuscript. All authors read and approved the final manuscript.

**Acknowledgments**

The author would like to thanks the DHS program for providing the data.

**References**

1. UNAIDS. 2017. Women and Girls and HIV. Geneva, Switzerland2018.
2. World Health Organization (WHO). 2019. Maternal, newborn, child and adolescent health. HIV and youth. [Available from: [https://www.who.int/maternal_child_adolescent/topics/adolescence/hiv/en/.](https://www.who.int/maternal_child_adolescent/topics/adolescence/hiv/en/)
3. UNAIDS. 2018. State of the epidemic. Number of AIDS-related deaths, global, 1990–2017 and 2020 target.
4. STOP AIDS. 2016. ‘Adolescents and young people and HIV’[pdf] [Available from: [https://stopaids.org.uk/wp/wp-content/uploads/2017/06/STOPAIDS-Factsheet-Adolescents-and-young-people-and-HIV.pdf.](https://stopaids.org.uk/wp/wp-content/uploads/2017/06/STOPAIDS-Factsheet-Adolescents-and-young-people-and-HIV.pdf)
5. UNAIDS. 2016. Ending the AIDS epidemic for adolescents, with adolescents. A practical guide to meaningfully engage adolescents in the AIDS response. Geneva, Switzerland.
6. Unicef. 2019. Adolescent HIV prevention 2018 [Available from: [https://data.unicef.org/topic/hivaids/adolescents-young-people/.](https://data.unicef.org/topic/hivaids/adolescents-young-people/)
7. World Health Organazation (WHO). Consolidated guideline on sexual and reproductive health and rights of women living with HIV. 2017.
8. UNAIDS. Women and HIV — A spotlight on adolescent girls and young women. 2019

[(https://www.unaids.org/sites/default/files/media_asset/2019_women-and-hiv_en.pdf](https://www.unaids.org/sites/default/files/media_asset/2019_women-and-hiv_en.pdf)).

9. WHO/UNAIDS. WHO, UNAIDS STATEMENT ON HIV TESTING SERVICES: NEW OPPORTUNITIES AND ONGOING CHALLENGES. 2017.
10. World Health Organazation (WHO). Statement on HIV testing services. HIV/AIDS. 2019 [Available from: [https://www.who.int/hiv/topics/vct/hts-new-opportunities/en/.](https://www.who.int/hiv/topics/vct/hts-new-opportunities/en/)
11. ETHIOPIAN FEDERAL HIV/AIDS PREVENTION AND CONTROL OFFICE. HIV/AIDS STRATEGIC PLAN2015-2020 IN AN INVESTMENT CASE APPROACH. 2014.
12. National Population Commission (NPC) [Nigeria] and ICF International. Nigeria Demographic and Health Survey 2013. Abuja, Nigeria, and Rockville, Maryland, USA: NPC and ICF International.2014.
13. National Institute of Statistics of Rwanda (NISR) [Rwanda] MoHMR, and ICF International. 2015. Rwanda Demographic and Health Survey 2014-15. Rockville, Maryland, USA: NISR, MOH, and ICF International.
14. Uganda Bureau of Statistics (UBOS) and ICF. 2018. Uganda Demographic and Health Survey 2016. Kampala, Uganda and Rockville, Maryland, USA: UBOS and ICF.
15. National Council for Population and Development (NCPD) [Kenya] and ICF International. Kenya Demographic and Health Survey 2014. Nairobi, Kenya, and Rockville, Maryland, USA: NPC and ICF International2015.
16. K Peltzer, G Matseke. Determinants of HIV testing among young people aged 18 – 24 years in South Africa. African Health Science 2013;13.

17. Michael J.Mahande, Rune N. Phimemon, Habib O. Ramadhani1. Factors associated with changes in uptake of HIV testing among young women (aged 15–24) in Tanzania from 2003 to 2012. BMC Infectious Diseases of poverty. 2016;5(11)(92).
18. Ibitola O. Asaolu, Jayleen K. Gunn, Katherine E. Center, Mary P. Koss, Juliet I. Iwelunmor, Ehiri JE. Predictors of HIV Testing among Youth in Sub-Saharan Africa: A Cross-Sectional Study. PLoS One 2016.
19. Patou Masika Musumari, Arunrat Tangmunkongvorakul, Kriengkrai Srithanaviboonchai, Sawang Yungyuankul, Teeranee Techasrivichien, S. Pilar Suguimoto, et al. Prevalence and Correlates of HIV Testing among Young People Enrolled in Non-Formal Education Centers in Urban Chiang Mai, Thailand: A Cross-Sectional Study. PLoS One 2016.
20. Mamuka Djibuti, Tamar Zurashvili, Tamar Kasrashvili, Carla J Berg. Factors associated with HIV counseling and testing behavior among undergraduates of universities and vocational technical training schools in Tbilisi, Georgia. BMC Public Health 2015;15(427).
21. Menon AJ, Mwaba SO, Thankian K. Determinants of HIV Testing Among Sexually Active Young People in Zambia. AFRREV IJAH: An International Journal of Arts and Humanities. 2017;6(4):130-42.
22. Mahande MJ, Phimemon RN, Ramadhani HO. Factors associated with changes in uptake of HIV testing among young women (aged 15–24) in Tanzania from 2003 to 2012. Infectious diseases of poverty. 2016;5(1):92.
23. Nwachukwu CE, Odimegwu C. Regional patterns and correlates of HIV voluntary counselling and testing among youths in Nigeria. African journal of reproductive health. 2011;15(2).
24. Ibrahim M, Ipadeola O, Adebayo S, Fatusi A. Socio-demographic determinants of HIV counseling and testing uptake among young people in Nigeria. International Journal of Prevention and Treatment. 2013;2(3):23-31.
25. Oginni A, Obianwu O, Adebajo S. Socio-demographic Factors Associated with Uptake of HIV Counseling and Testing (HCT) among Nigerian Youth. AIDS research and human retroviruses. 2014;30(<https://www.researchgate.net/publication/267727859>).
26. Ethiopian Ministry of Health. Health Sector Transformation Plan2015/16 - 2019/20. 2015.
27. United Nation (NU). Sustainable development goal. 2015.
28. Girum T, Wasie A, Worku A. Trend of HIV/AIDS for the last 26 years and predicting

achievement of the 90–90-90 HIV prevention targets by 2020 in Ethiopia: a time series analysis. BMC infectious diseases. 2018;18(1):320.

29. Ethiopian Central Statistics Agency(ECSA). Ethiopian demographic health survey (EDHS). 2016.
30. MacPhail C, Pettifor A, Moyo W, Rees H. Factors associated with HIV testing among sexually active South African youth aged 15–24 years. AIDS care. 2009;21(4):456-67.
31. Mafigiri R, Matovu JK, Makumbi FE, Ndyanabo A, Nabukalu D, Sakor M, et al. HIV prevalence and uptake of HIV/AIDS services among youths (15–24 years) in fishing and neighboring communities of Kasensero, Rakai District, south western Uganda. BMC public health. 2017;17(1):251.
32. National HIV/AIDS Strategic Plan 2010–2015. 2010 [Available from: [http://www.nationalplanningcycles.org/sites/default/files/country_docs/Nigeria/hiv_plan_nigeria.pdf.](http://www.nationalplanningcycles.org/sites/default/files/country_docs/Nigeria/hiv_plan_nigeria.pdf)
33. Kyaddondo D, Wanyenze RK, Kinsman J, Hardon A. Home-based HIV counseling and testing: client experiences and perceptions in Eastern Uganda. BMC Public Health. 2012;12(1):966.
34. Damian Jeremia, Msuya S. HIV prevalence and factors associated with HIV testing among young people (15–24 years) in Tanzania: A secondary analysis of THMIS data. researchgate. 2015(<https://www.researchgate.net/publication/281964872>).
35. Jamison EA, Jamison DT, Hanushek EA. The effects of education quality on income growth and mortality decline. Economics of Education Review. 2007;26(6):771-88.
36. Molla G, Huruy A, Mussie A, Wondowosen T. Factors associated with HIV counseling and testing among males and females in Ethiopia: evidence from Ethiopian Demographic and Health Survey data. Journal of AIDS and Clinical Research. 2015;6(3).

37. Kristen Hibbett. 2018. Available from: [https://borgenproject.org/addressing-the-barriers-to-proper-health-care-in-ethiopia/.](https://borgenproject.org/addressing-the-barriers-to-proper-health-care-in-ethiopia/)
38. Teklehaimanot HD, Teklehaimanot A, Yohannes M, Biratu D. Factors influencing the uptake of voluntary HIV counseling and testing in rural Ethiopia: a cross sectional study. BMC public health. 2016;16(1):239.
39. Drinking and Risky Sexual Behavior [Internet]. 2015.
40. Ivona Pandrea, Kyle I. Happel, Angela M. Amedee, Gregory J. Bagby, Nelson S. Alcohol’s Role in HIV Transmission and Disease Progression. 2010(<https://www.ncbi.nlm.nih.gov/pmc/articles/PMC3860502/>).
41. FEDERAL MINSTRY OF HEALTH. [ETHIOPIAN]. 2017. NATIONAL GUIDELINES FOR COMPREHENSIVE HIV PREVENTION, CARE AND TREATMENT.
42. Caitlin E. Kennedy, Virginia A. Fonner, Michael D. Sweat, F. Amolo Okero, Rachel Baggaley, O’Reilly KR. Provider-Initiated HIV Testing and Counseling in Low- and Middle-Income Countries: A Systematic Review. 2014;17(<https://www.ncbi.nlm.nih.gov/pmc/articles/PMC3927322/>).
