# Supplementary material for: Factors associated with HIV testing among young females; further analysis of the 2016 Ethiopian demographic and health survey data
Source: PLoS One. 2020 Feb 11;15(2):e0228783. doi: 10.1371/journal.pone.0228783 (PMC7012428; doi:10.1371/journal.pone.0228783)
Supplement: Attachment — Submitted filename: Response to reviwers.docx [file pone.0228783.s003.docx]

Point by point response

| **S/N** | **Reviewers’ /editor’s comments** | | **Authors response** |
| --- | --- | --- | --- |
|  | **Editor’s comments** | |  |
| 1. | Typo in Reference 7, 10 correct spelling of "Organazation" | | Thank you for the comment. We correct the spellings of “organization” on the updated manuscript. (line number 35-and page numbers) |
| 2. | Ref 8, 9, 11, 14 - Please change from CAPS | | Thank you for the comment. We change CAPS on the updated manuscript. (line number 259 and page number 13) |
| 3. | Ref 16 - Not consistent with referencing guidelines. Has initial then surname. Please correct | | Thank you for the suggestion. We accept and amend the correction on the updated manuscript  (line number 310 and page number 13) |
| 4. | Ref 17, 18, 19, 20 is non-compliant with referencing guidelines. Please correct | | Thank you for the comment. We made correction and in line with the referencing guide line. (line numbers 314-322 and page number 14) |
| 5. | Ref 39: Who is the author? | | Thank you for the comment. We correct it “Hopkins J. Drinking and Risky Sexual Behavior 2015”  (line number 364 and page number 15) |
| **Reviewer 1** | | | |
| **Abstract section** | | | |
| 1. | Lacked quantifications of the problem, how low is low HIV testing in Ethiopia? | | Thank you for the comment. We quantified the problem specially in Ethiopia context  (line number 35 and page number 2 ) |
| 2. | Regarding the knowledge gap:   a. What are the advantages of us having this information? Who will benefit from the study being conducted and how? b. Limited information does not warrant a study to be conducted. The missing information has to be useful in some way to the study stakeholders. | | Thank you for the comment. We revised the introduction to show the importance of conducting the study. In the revision, we included beneficiaries of the study output. (line numbers 36-39 and page number 2) |
| 3. | Would be better to mention the age range. Who is considered to be a young woman for this study? 15-19 years? Or 15-24? | | Thank you for the suggestion. In this study young means those women’s whose age 15-24 years old. And this is included in the revised version. (line numbers 35, 37, 41-42 and page number 2) |
| 4. | The conclusion summarized the results without any further recommendations.   a. Recommendations are needed to show the implication of the study findings either to policy and/or to practice. | | Thank you for the suggestion. Based on your comment we incorporate recommendation on conclusion section.  (Line numbers 56-58 and page number 2) |
| **Introduction section** | | | |
| 1. | Throughout the entire background, entire paragraphs had only one citation on the last sentence. Please revisit citations. | | Thank you for the comment. We revise and made correction on the updated manuscript  (line numbers 65- 107 and page numbers 3and 4) |
| 2. | It is still unclear who is a “young woman”. It is informative for the reader to have an age range earlier on in the paper. | | Thank you for the suggestion. A young woman means, a woman’s whose age between 15 -24 years old.  (line numbers 65-67,72,81 and page number 3) |
| 3. | For the paragraph concerning previously identified significant predictors;  It would be more informative to mention the exact levels/categories which were found to be significant predictors e.g. Rural/urban? Which category was significant?  It would also be informative to mention whether they are predictors of higher/lower odds of HIV testing. | | Thank you for the comment. Based on the comment we clearly mentioned the level and categories significantly associated with HIV testing.  (line numbers 86-94 and page numbers 3 and 4) |
| 4. | And thereafter identifying the factors, how would the results be used? After these bottle-necks have been identified. | | Thank you for the comment. The ministry of health and other organizations working on HIV may use to strengthen HIV prevention activities. This idea is included in the revised section of the manuscript.  (line numbers 103-107 and page number 4) |
| **Methodology section** | | | |
| 1. | Why only include women who were sexually active in the past 12 months into the study? Please provide an elaboration.   - The risk of HIV does not change regardless of the time since last sexual intercourse.   Wouldn’t a more appropriate exclusion criteria be women who NEVER had sexual intercourse? | | Thank you for the comment. We included women who had history of sexual intercourse with in the last 12 months. This to reduce recall bias. |
| 2. | For the outcome definition;  Why the focus on those who tested in past 12 months?  Why not use if she EVER tested for HIV?  Why are those who tested before 12 months considered as if they have never tested and coded as 0? | | Thank you for the comment. We limited the analysis HIV testing for the last 12 months to see the most recent trend. We feel that the most recent information is more important than the earlier ones for HIV prevention. If sexually active, the testing information in the last year is more informative than history of testing before four years. In addition, most of the variables, for example, characteristics of women are at the time of survey. Since the data collection was cross-sectional, events that happened years before the time of survey may have different implications. For example, if we take age, and include young women who were tested 5 years before, the information about age will be quite different. |
| 3. | How was HIV knowledge measured? | | Thank you for the suggestion. This variable was not included in the analysis. Now it is deleted. (line number 128 and page number 5) |
| 4. | Did you consider using alternatives to logistic regression if you identified the outcome of interest to be common? >10% prevalent. | | Thank you for the comment. We did not consider because the outcome variable is binary. We feel that this is the best option. |
| 5. | “Bivariate regression analysis was done to examine associations between contraceptive use and the selected predictor variables”;  Does this study measure contraceptive use OR HIV testing? | | Thank you for the comment and we would like to say sorry for the silly mistake we made. It was to say “HIV testing”. Finally we made amendment on the updated manuscript (line number 135 and page number 5) |
| 6. | “In addition, correlation among predictor variables was assed using variance inflation factor before recruiting variables to the final mode.”   - What do you mean? Is it correlation or collinearity? | | Thank you the comment and We would like to say sorry again for made this silly mistake using “correlation” instead of “collinearity”. Finally we made amendment on the updated manuscript (line number 137 and page number 5) |
| **Result section** | | | |
| 1. | For every table caption, please include N=??? | | Thank you for the suggestion. Based on the comment we included N in the entire table in the updated manuscript. |
| 2. | For every variable in the tables with category “other”, please provide an explanation on who/what “other” means. | | Thank you for the suggestion. Based on the recommendation we provided footnote explanation what “other” means. |
| 3. | Did you consider life time sexual partners? Or those in the past 12 months? Please make clear. | | Thank you for the comment. In this analysis we considered “life time sexual partners” not “those in the past 12 months”. |
| 4. | What is chat? Concerning the variable “chewed chat” | | Thank you for the constructive comment. Chat is a flowering plant native to the horn of Africa and Arabian Peninsula commonly called as “khat” but it’s commonly called as “chat” in our country Ethiopia. But to make it familiar we change “chat” in to “khat” in the updated manuscript. |
| 5. | The percentages presented in Table 3 are column percentages,  Which means the interpretations should be “among those tested for HIV, 26.22% were aged 15-19”.  Please revise the interpretation provided above. | | Thank you for the constructive comments. The interpretation is corrected now. The data for those aged 15 – 19 is presented in the table. |
| **Discussion**  **Please apply these for the entire discussion section;** | | | |
| 1. | For each finding, it would be more informative to report numbers/findings that these previous studies reported. It would be informative to mention them in brief so the reader may compare. | | Thank you for the constructive comment. Based on your recommendation we incorporated the findings of each study on the updated manuscript.  (line numbers 204-208 and page number 10) |
| 2. | What is the implication of each study finding? Are you recommending for the Ethiopian government to adopt a similar strategy to increase HIV testing as Uganda? | | Thank you for the constructive comment. Based on the recommendation we incorporated the implications of each finding on the updated manuscript.  (line numbers 213 -2014 and page number 10) |
| 3. | Discussing only similarities/differences of study findings is not enough. You need to go a step further and show the implications of this study findings to either policy or to practice. | | Thank you for the constructive comment. Based on the recommendation we included the implications of each finding on the updated manuscript.  (line numbers 220 – 222, 226-227, 242-243 and page number 10) |
| **Strength and limitation** | | | |
| 1. | This section is missing from the paper | | Thank you for the constructive comment. We included the limitation and strength in the updated manuscript.  (line numbers 244-247 and page number 11) |
| **Conclusion and Recommendation section** | | | |
| 1. | Please provide recommendations basing on the results of this study. How can these findings be applied to benefit the stakeholders of the study? The women, policy makers, government officials? | | Thanks for the constructive comment. Based on your recommendation we included recommendation in the updated manuscript.  (line numbers 252-255 and page number 12) |
| Reviewer 2 | | | |
|  | | Abstract | |
| 1. | | Few grammatical errors exist and one statements (e.g ….. those who ever had alcohol (AOR 1.46; 95% (CI; 1.09, 1.98)) and young women who visited health facility (AOR 1.78; (95% CI; 1.36, 2.32)) higher odds of being tested for HIV.) has a word missing | Thank you for the comment. All grammatical errors and missing statements are corrected in the revised manuscript.  (line numbers 49-51 and page number) |
|  | | Background | |
| 2. . | | Paragraph 3, last statement needs further elaboration to put context. It is unclear as to which population is represented by the 30 % of people living with HIV. | Thank you for the comment. It is revised and made clear now.  (line numbers 78-79 and page number 3) |
| 3. | | Citations need to be specific to the statements rather than lumping all the references to the last statement of the paragraph. | Thank you for the constructive comments. The citations are revised the revised manuscript. |
| 4. | | The last paragraph of introduction indicates that there are few studies, on factors associated with HIV testing among young women in Ethiopia. Unless the gap is identified in those studies, the current study is unjustifiable. The authors may need to present what those studies found and their gaps to justify why the current study is needed. | Thank you for the suggestion, the paragraph is revised to show the existing gaps and the importance of the study  (line numbers 103- 107and page number 4) |
| 5. | | The authors may need to revise the whole introduction section and correct the grammatical, spelling, and space errors. Methods and Materials | Thank you for the comment. We corrected the grammatical, spelling and spacing errors in the revised manuscript. |
|  | | Methods | |
| 6. | | Statistical analysis needs to be described clearer. The authors indicate that “Bivariate regression analysis was done to examine associations between contraceptive use and the selected predictor variables. Contraceptive use was not listed as an outcome variable in the above section. Authors may need to check if this is correct. | Thank you for the comment. We understood that we made editing errors. The dependent variable was HIV testing, not contraceptive use. Now, we revised it. (line numbers 134- 137 and page number 5) |
| 7. | | The procedure for weighting the data to account for non-response and disproportionate sampling need to be transparently described. | Thank you for the comment. EDHS takes stratified random sampling technique. Samples taken from some regions may be high and in other regions low. Therefore, DHS recommend data weighting to reduce the effect of over sampling or under sampling. The details can be find in the DHS website. |
| Results | | | |
| 8. | | The results are well written; however, the authors may consider making the results more concise. It is possible to fuse tables 1 and 3; and 2 and 3 by considering that the numbers in table 3 are subsets of Tables 1 and 2. | Thank you for the comment. We merged table 1 and 2. |
| 9. | | The authors may also need to indicate which variables were adjusted in the multivariate model, what criteria were used for selecting such variables for adjusting and/or justification. | Thank you for the comment. All variables were which were not multicollinear were included to the multivariate logistic regression model |
| 10. | | The data for crude odds ratio for rural residency in table 4 is missing | Thank you for the comment. It is included in the revised version. |
| 11. | | The authors may also want to reformat the Table 4 so that numbers separated by comma are spaced. | Thank you for the comment. It is formatted now. |
| Discussion | | | |
| 12. | | The discussion has interpreted and compared the findings with the previous studies. However, the authors may need to restructure the discussion a bit, so they begin the section by summarizing what they found and later discussing the results. | Thank you for the constructive comment. The discussion is revised now. |
| 13. | | Discussion of the methodological strengths and weaknesses/limitations of their study is missing | Thank you for the comment. It is included at the end of the discussion section in the revised manuscript. (line numbers 244-247 and page number 11 ) |
| 14. | | Although they found the factors associated with HIV testing among sexually active young women in Ethiopia, their discussion needs to translate and discuss the findings by relating to the real issues among young women in Ethiopia. | Thank you for the comment.  We tried to translate the findings to the Ethiopian context |
| 15. | | To improve the success of the HIV counseling and testing program, the authors need to indicate the implications for practice and further research. | Thank you for the comment. We included recommendation in the revised section of the manuscript. |
