# Supplementary material for: Factors associated with HIV testing among young females; further analysis of the 2016 Ethiopian demographic and health survey data
Source: PLoS One. 2020 Feb 11;15(2):e0228783. doi: 10.1371/journal.pone.0228783 (PMC7012428; doi:10.1371/journal.pone.0228783)
Supplement: Attachment — Submitted filename: piont by piont response.docx [file pone.0228783.s004.docx]

Point by point response

1. The manuscript requires significant copyediting before acceptance.

*Thank you for your suggestion. For addressing the issue we consulted senior public health staff and language professors in my university. We also used online softwares, specifically grammerly and scribens (check for correctness of spellings).*

2. Title page: Correspondent should read corresponding

*Thank you for your comment. Based on your comment we made amendment*

3. Abstract Line 5. low HIV testing and counseling service use better written low utilization of HIV testing and counseling service

*Thank you for your suggestion. Based on your suggestion we rewrite it.*

4. Introduction: Line 1 5,000 people were infected should be 5,000 people are infected

*Thank you for your suggestion. Based on your suggestion we rewrite it.*

5. Line 3 disproportionally should be disproportionately

*Thank you for your comment. We correct the spelling error on the updated manuscript.*

6. Intro 2nd para Line 1 and 2 "early life of transmitted from mother to child …… needs to rephrase.

*Thank you for your suggestion. Based on your suggestion we rewrite it.*

7. There are many more in the narrative

*Thank you for your comment. Based on your comment we revise the rest parts of the document.*
